# Normothermia after decompressive surgery for space-occupying middle cerebral artery infarction: a protocol-based approach

**DOI:** 10.1186/s12883-017-0988-x

**Published:** 2017-12-04

**Authors:** Jan Rahmig, Matthias Kuhn, Hermann Neugebauer, Eric Jüttler, Heinz Reichmann, Hauke Schneider

**Affiliations:** 1 Department of Neurology, University Hospital, Technische Universität Dresden, Dresden, Germany; 2 Institute of Medical Informatics and Biometry, Technische Universität Dresden, Dresden, Germany; 3 Department of Neurology, University of Ulm, Ulm, Germany; 4 Department of Neurology, Ostalb-Klinikum Aalen, Aalen, Germany; 5 Department of Neurology, Klinikum Augsburg, Augsburg, Germany

**Keywords:** Stroke, Space-occupying infarction, Decompressive surgery, Hemicraniectomy, Temperature management, Normothermia

## Abstract

**Background:**

Moderate hypothermia after decompressive surgery might not be beneficial for stroke patients. However, normothermia may prove to be an effective method of enhancing neurological outcomes. The study aims were to evaluate the application of a pre-specified normothermia protocol in stroke patients after decompressive surgery and its impact on temperature load, and to describe the functional outcome of patients at 12 months after treatment.

**Methods:**

We analysed patients with space-occupying middle cerebral artery (MCA) infarction treated with decompressive surgery and a pre-specified temperature management protocol. Patients treated primarily with device-controlled normothermia or hypothermia were excluded. The individual temperature load above 36.5 °C was calculated for the first 96 h after hemicraniectomy as the Area Under the Curve, using °C x hours. The effect of temperature load on functional outcome at 12 months was analysed by logistic regression.

**Results:**

We included 40 stroke patients treated with decompressive surgery (mean [SD] age: 58.9 [10.1] years; mean [SD] time to surgery: 30.5 [16.7] hours). Fever (temperature > 37.5 °C) developed in 26 patients during the first 96 h after surgery and mean (SD) temperature load above 36.5 °C in this time period was 62,3 (+/− 47,6) °C*hours. At one year after stroke onset, a moderate to moderately severe disability (modified Rankin Scale score of 3 or 4) was observed in 32% of patients, and a severe disability (score of 5) in 37% of patients, respectively. The lethality in the cohort at 12 months was 32%. The temperature load during the first 96 h was not an independent predictor for 12 month lethality (OR 0.986 [95%-CI:0.967–1.002]; *p* < 0.12).

**Conclusions:**

Temperature control in surgically treated patients with space-occupying MCA infarction using a pre-specified protocol excluding temperature management systems resulted in mild hyperthermia between 36.8 °C and 37.2 °C and a low overall temperature load. Future prospective studies on larger cohorts comparing different strategies for normothermia treatment including temperature management devices are needed.

**Electronic supplementary material:**

The online version of this article (10.1186/s12883-017-0988-x) contains supplementary material, which is available to authorized users.

## Background

Early decompressive hemicraniectomy can increase survival and improve functional outcome in patients with space-occupying middle cerebral artery (MCA) infarction [1–3]. The swelling of infarcted brain tissue reaches its maximum between day 2 and 5 after stroke onset [4]. Infarction edema leading to cerebral herniation is the main cause of early, intra-hospital deaths in surgically treated patients [2, 3]. Conservative treatment options, e.g. osmotherapy or deep sedation with barbiturates, are used to treat infarction edema, although data from randomized trials supporting their use are lacking [5].

Fever occurs in about half of all stroke patients and is associated with worse functional outcome of affected patients [6, 7]. Fever control and treatment of the underlying causes are well-established parts of routine clinical practice, but sufficient data are lacking to support the use or the type and timing of fever control in stroke patients [8, 9]. Temperature management can include antipyretics (e.g. acetaminophen, metamizole), physical applications (e.g. wet blankets), and the use of temperature management systems (TMS), either as endovascular or body surface devices [10].

Normothermia in patients with space-occupying infarction, especially within the first five days after hemicraniectomy, could avoid brain volume increase and critical rise of intracranial pressure that might be associated with elevated body temperature. However, there are no conclusive studies that demonstrate that normothermia is associated with improved functional outcomes in these patients.

Controlled moderate hypothermia, with target temperatures between 33 °C and 35 °C, has been evaluated as a treatment option for ischemic stroke including space-occupying infarction [11–16]. Recent studies including a randomized controlled trial suggest that hypothermia in addition to hemicraniectomy might not be beneficial for patients with malignant MCA infarction, partially due to a higher rate of adverse events in cooled patients [17, 18].

Controlled normothermia to prevent fever was suggested as a promising approach to improve functional outcome compared to strategies that are reactive to temperature elevations. Detailed data on temperature course of surgically treated stroke patients managed with “reactive” normothermia protocols are not available. This limits the ability to calculate e.g. the sample sizes for randomized trials comparing normothermia strategies [19].

The aims of our study are therefore: 1) to describe in detail the individual temperature course and temperature load above 36.5 °C in stroke patients during the first 96 h after hemicraniectomy, using a pre-specified normothermia protocol excluding temperature management systems, and 2) to evaluate the functional outcome at 12 months after treatment.

## Methods

### Study design

This is a retrospective observational cohort study. Functional outcome at 6 and 12 months was assessed prospectively by structured telephone interviews: 1) for patients included in our local registry for space-occupying MCA infarction of older patients (age < 60 years), and 2) for patients that were included in randomized trials at our center (1 and 2: *n* = 26). For the remaining patients (*n* = 14), we evaluated functional outcome using hospital and rehabilitation reports stating the modified Rankin Scale score, and / or by a structured telephone interview to retrospectively assess the modified Rankin Scale score if hospital and rehabilitation reports were insufficient. Functional outcome was assessed for all patients of the cohort by study personal or investigators not involved in patient care and before study specific data analyses were performed.

### Patient selection

We identified stroke patients treated with hemicraniectomy between November 2011 and June 2016, searching: a) our research registry that includes patients over 60 years old with space-occupying MCA infarction and hemicraniectomy (*n* = 15), b) local patient logs of completed randomized trials of hemicraniectomy (DESTINY II, *n* = 3; DEPTH-SOS, *n* = 8), and c) our electronic hospital information system (*n* = 14).

The inclusion criteria were: a) space-occupying ischemic infarction in the MCA territory, b) hemicraniectomy, c) temperature management according to our pre-specified protocol (below) and d) no treatment with temperature management systems to induce normothermia or hypothermia.

### Stroke treatment and intensive care

Patients were treated according to current European stroke guidelines. Stroke treatment included systemic thrombolysis and/or endovascular therapy if indicated by the treating physician. According to our institutional standard for treatment of space-occupying infarction, decompressive surgery should be performed within 48 h after stroke onset (or time of last seen well), if the following criteria have been fulfilled: a) clinical signs of MCA infarction, b) infarction >2/3 of the MCA territory, detected on cerebral CT or MRI, c) NIHSS >15 (non-dominant hemisphere) or NIHSS >20 (dominant hemisphere), and d) reduced level of consciousness. Postoperatively, patients were transferred sedated, intubated, and mechanically ventilated to our ICU. Neuromonitoring included intracranial pressure (ICP) monitoring using an ipsilaterally inserted ICP probe. A cerebral CT was performed 24 (12–36) hours after hemicraniectomy. Patients with progressive edema were maintained deeply sedated (midazolame, propofol) and received osmotherapy with mannitol if ICP was elevated above 15 mmHg. Furthermore, the cerebral perfusion threshold was defined as 70 mmHg. ICP rescue interventions were osmotherapy with hypertonic sodium solutions and barbiturate sedation.

### Pre-specified temperature management protocol

Normothermia was defined as a body core temperature of 36.5 °C, measured by a bladder catheter. Temperature was measured continuously and documented automatically every hour in the electronic patient management system. Hyperthermia >37.5 °C was treated primarily with acetaminophene as a short infusion (1 g). Patients with persistent hyperthermia after 2 h received further acetaminophene infusions (maximum 4 g/d). Non-responders to acetaminophene received metamizole as a short infusion (1 g) and/or cold water wrappers on their arms, trunk and legs.

Infection monitoring consisted of a daily clinical evaluation; daily laboratory testing of blood count, C-reactive protein, and/or procalcitonin; and a chest X-ray every second day for ventilated patients. Further diagnostic procedures to evaluate infections (e.g., urine analysis, cultures, ultrasound, CT) were performed if local or systemic signs of infections were present. Antibiotic therapy was applied according to our institutional standard if indicated.

### Data management

Data on baseline patient characteristics and hospital treatment were retrieved from electronic patient files (ICM®, Dräger; ORBIS®, Agfa). Vital paramaters including body core temperature were measured continuously and transferred hourly to electronic patient files (ICM®, Dräger). Individual temperature data were retrieved as electronic files for analysis and stored electronically. All data were checked for completeness and consistency by two investigators.

### Temperature load

Mean time from symptom onset to hemicraniectomy was expected to be between 24 and 36 h. We selected an evaluation period of 96 h postoperatively to fully cover the critical phase of brain edema, usually observed between days 2 and 5 after infarction.

Temperature load (TL) was defined as the sum of the products of body temperature above the temperature level of 36.5 °C (defined as normothermia) and time (°C * hours), calculated for each hour during the postoperative 96 h. TL therefore corresponds to the area under the temperature curve above a temperature of 36.5 °C. The TL for each patient was calculated in two steps by the following formulas (condition: *t*_*i*_ > 0) (t = temperature; h = hours; TL = temperature load) (11):

1 $$ \overline{t_i}=\frac{t_i+{t}_{i+1}}{2}-36.5 $$

2 $$ \mathrm{TL}={\sum}_{i=1}^{96}\overline{t_i}\times 1h $$

For subgroup analyses we calculated the median temperature load above the temperature level of 36.5 °C of the cohort and divided the cohort into two patient groups, 1) low temperature load and 2) high temperature load, according to the median TL.

The temperature load above the temperature level of 37.5 °C was calculated accordingly for each patient for the first 96 h after decompressive surgery.

### Statistical analysis

We calculated frequencies, means with standard deviations (SD), and median values with inter-quartile ranges (IQR) for the whole cohort and two subgroups. The two subgroups were defined according to the median temperature load of the cohort (low temperature load group [*n* = 20] and high temperature load group [*n* = 20]). Student’s t-test was used for normally distributed data; the Mann-Whitney U test was used for skewed continuous data and ordinal data; and Fischer’s exact test was used for categorical data.

Univariate regression analyses were performed to evaluate the impact of clinically relevant patient and treatment characteristics, including temperature load and temperature deviation, on mortality at 12 months. Explorative multivariate regression analyses with 3 factors were calculated, including the factors temperature load (metric or dichotomized according to the median temperature load of the cohort), temperature deviation, age, sex, affected hemisphere, stroke severity (NIHSS), or diameter of hemicraniectomy. Hosmer-Lemeshow tests were performed for all models.

We used the software packages SPSS, version 23 (IBM Corp.; USA), and R, version 3.2.3 (R Foundation; http://www.r-project.org/), for statistical analyses.

### Ethical statement

The study was approved by our institutional review board (Ethikkommission an der TU Dresden; IRB00001473; EK125042012, EK204062013).

### Informed consent

Due to the retrospective study design, no informed consent was needed to include patients in the study. An informed consent was obtained from patients or their legal representatives, if functional outcome was evaluated retrospectively by a structured telephone interview.

## Results

### Patient cohort: Characteristics, treatment, and functional outcome

The study cohort consisted of 40 patients with space-occupying MCA infarction treated with decompressive surgery. Table 1 shows baseline and treatment characteristics of the patients. Mean patient age was 58.9 years and mean time to surgery was 30.5 h (median 29.50 [IQR 18.5–35] hours). Thirty patients of the cohort were transferred from rural hospitals to the stroke centre (e.g. for endovascular therapy, decompressive surgery).

**Table 1:** Baseline and treatment characteristics

	All patients (*n* = 40)	Low temperature load patients (*n* = 20)	High temperature load patients (*n* = 20)	*p* value
Age, years, mean (SD)	58.9 ± 10.1	59.0 ± 10.6	58.9 ± 9.9	0.976
Sex (female), n (%)	19 (47.5)	10 (50.0)	9 (45.0)	>0.99
Arterial hypertension, n (%)	25 [62.5]	13 [65)	12 [60]	>0.99
Hyperlipidemia, n (%)	11 [27.5]	7 [35]	4 [20]	0.480
Diabetes mellitus, n (%)	14 [35]	7 [35]	7 [35]	1.0
Atrial fibrillation, n (%)	10 [25]	5 [25]	5 [25]	1.0
Transfer from rural hospital to stroke center, n (%)	30 [75]	16 [80]	14 [70]	0.716
NIHSS score (admission), median (IQR)	32 [20–32]	28 (20.25–32)	32 (18–32)	0.695
Dominant hemisphere, n (%)	22 (55.0)	14 (70.0)	8 (40.0)	0.111
Intravenous rtPA, n (%)	20 [50]	7 [35]	13 [65]	0.113
Endovascular therapy, n (%)	8 [20]	2 [10]	6 [30]	0.235
Symptom onset to hemicraniectomy, h, mean (SD)	30.5 (16.7)	31.6 (22.2)	29.4 (10.3)	0.695
Diameter of hemicraniectomy, cm, median (IQR)	12.9 (12.1–13.4)	13.0 (12.1–13.5]	12.8 (12.0–13.3)	0.867
Period of 96 h after surgery				
Leucocytosis, n (%)	36 (90)	17 (85)	19 (95)	0.605
Antibiotics, n (%)	40 (100)	20 (100)	20 (100)	NA
Antipyretics, n (%)	35 (87.5)	15 (75)	20 (100)	*0.024*
One antipyretic, n (%)	15 (37.5)	8 (40)	7 (35)	
Two antipyretics, n (%)	20 (50)	7 (35)	13 (65)	
Acetaminophen, g, mean (SD)	2.95 (2.2)	2.4 (2.5)	3.5 (1.8)	*0.044*
Metamizole, g, mean (SD)	1.38 (1.9)	1.05 (1.85)	1.7 (2)	0.132
Temperature load 96 h, >36.5 °C; °C x h, mean (SD)	62.3 (47.6)	20 (19.2)	104.6 (22.4)	*< 0.001*
Temperature load 96 h, >37.5 °C; °C x h, mean (SD)	14.3 (15.4)	3.8 (7.3)	24.68 (14.4)	*< 0.001*
Patients with *T* > 37.5 °C, n (%)	26 (65)	12 (60)	14 (70)	0.741

*T* temperature, *SD* standard deviation, *NA* not applicable

All *p* values in italics are below 0.05

**Fig. 1:**
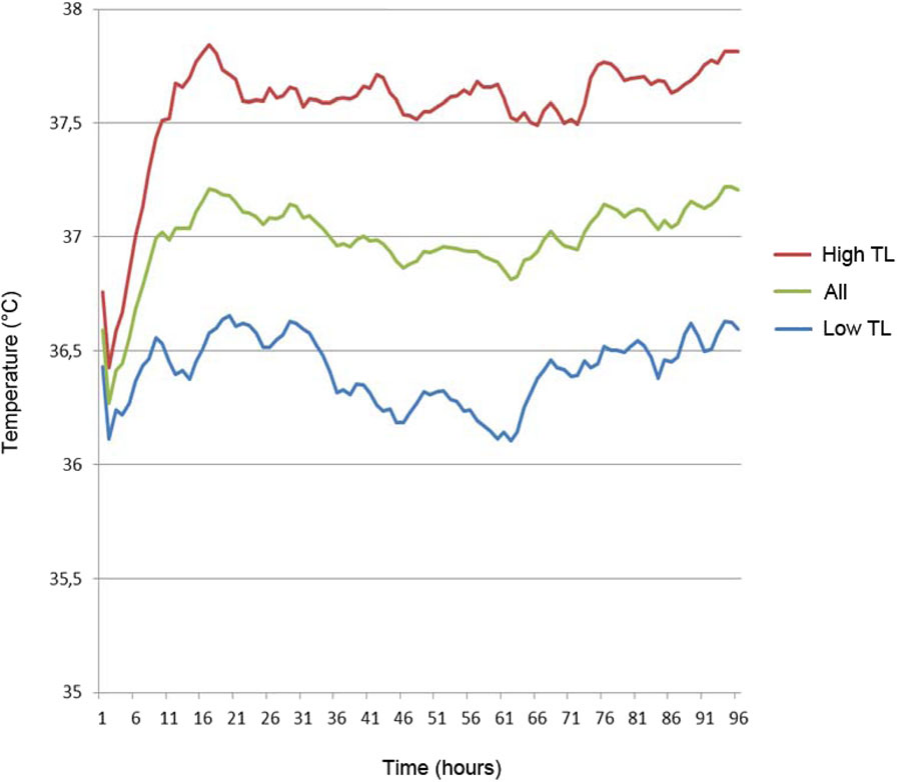
Body temperature course within 96 h after hemicraniectomy. Mean body temperature of the entire cohort (All), the low temperature load (Low TL) group, and the high temperature load (High TL) group, calculated for the pre-operative temperature and each hour postoperatively within the observational period

Postoperative mean body temperature course of the cohort is illustrated in Fig. 1, presenting the first 96 h after hemicraniectomy. Patients left the operating room mildly hypothermic and with a slightly lower body temperature compared to the pre-operative temperature. After transfer to the ICU, patients showed a moderate temperature rise within 24 h, peaking between 37.0 °C and 37.5 °C. In the following time period, body temperature remained widely stable between 36.8 °C and 37.2 °C. Individual temperature courses of patients with lowest and highest temperature deviations are presented in Additional file 1: Figure S1.

A body temperature above 37.5 °C was observed in 26 of 40 patients during the observation period. Mean temperature load above 37.5 °C in the cohort was 14.3 °C * hours during the first 96 h after hemicraniectomy. Severe fever above 39.0 °C was seen in only one patient and present for less than 2 h. Antipyretics were given to 34 patients; in 8 of these 34 patients, antipyretic drugs were given at body temperatures below 37.5 °C (36.7 °C – 37.4 °C). Fourteen patients received one antipyretic, and 20 patients received two antipyretics. The mean dose of acetaminophene, used as the first-line antipyretic, was 2.95 g during the 96-h-period. A leucocytosis was detected in 36 patients, and all patients received antibiotic therapy at some time during the observational period.

**Fig. 2:**
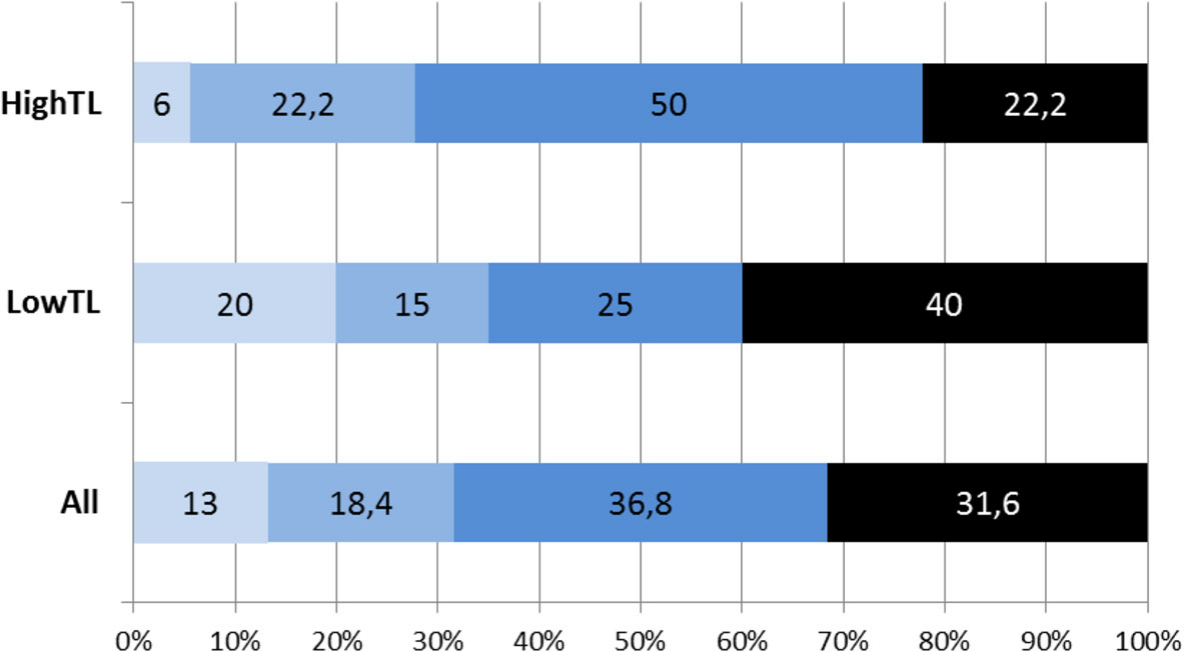
Functional outcome at 12 months on the modified Rankin Scale (mRS). mRS 0: no symptoms; mRS 1: no significant disability; mRS 2: slight disability and inability to carry out all pre-stroke activities; mRS 3: moderate disability, but being able to walk without personal assistance or a wheelchair; mRS 4: moderate to severe disability; needs assistance to attend to own bodily needs; unable to walk without assistance; mRS 5: severe disability; requires constant attention and care; bedridden; mRS 6: death

Intrahospital lethality of the cohort was 18% by 6 months and 32% by 12 months. Functional outcome according to the modified Rankin scale (mRS) score at 12 months is shown Fig. 2. For two patients of the high TL group, long term functional outcome could not be assessed.

### Comparison: Patients with low temperature load vs. high temperature load

We grouped patients according to their temperature load above 36.5 °C (area under curve, AUC; °C * hours), calculated for postoperative 96 h, into low TL and high TL patients using the median temperature load as the cut-off (*n* = 20 patients in each group). The low TL group had a mean temperature load of 20 (±19.2)°C*hr compared to 104.6(±22.4) °C*hr in the high TL group (*p* < 0.001).

Baseline and treatment characteristics of both patient groups are presented in Table 1. Cardiovascular risk factors were observed in comparable frequencies in both groups. Stroke severity on admission was slightly lower in the low TL group compared to the high TL group (median NIHSS score 28 vs. 32). The dominant hemisphere was affected more frequently in patients of the low TL group compared to the high TL group (70% vs. 40%). Patients with high TL had received intravenous rtPA and/or endovascular therapy more frequently compared to patients with low TL. The median diameter of hemicraniectomy was comparable in the two groups (low TL group: 13.0 cm vs. high TL group: 12.8 cm).

Mean body temperature courses of both groups are depicted in Fig. 1. Preoperative temperatures did not differ in the two groups. In the low TL group, a mostly normothermic course around 36.5 °C was observed during the 96 h period. Patients in the high TL group showed a temperature increase of about 1.5 °C within the first 24 h after hemicraniectomy and remained mildly to moderately hypertherm between 37.5 °C and 38.0 °C thereafter.

Treatment of hyperthermia with antipyretics was performed more often in the high TL group, and accordingly, the mean acetaminophene dose was higher in this group (low TL 2.4 g vs. high TL 3.5 g). All patients received antibiotic therapy. The length of the hospital stay was comparable in both groups.

The hospital mortality was not statistically significant different between the low TL and the high TL groups (30% vs. 5%; *p* = 0.09, Fisher’s exact test). (Table 2). The long-term functional outcome at 12 months after hemicraniectomy (Fig. 2) was not different in the two groups: mRS score 0–3: low TL 20% vs. high TL 5.6% (*p* = 0.34); mRS score 0–4: low TL 35% vs. high TL 27.8% (*p* = 0.74); The case fatality rate at 12 months was 40% in the low TL group and 22% in the high TL group (*p* = 0.31).

**Table 2:** Mortality and functional outcome

	All patients (*n* = 40)	Low temperature load patients (*n* = 20)	High temperature load patients (*n* = 20)	*p* value
Lethality, in-hospital, n (%)	7 (17.5)	6 (30)	1 (5)	0.09
Lethality at 12 months, n (%)	12 (31.6)*	8 (40)	4 (22.2)**	0.31
mRS 0–3 at 12 months, n (%)	5 (13.2)*	4 (20)	1 (5.6)**	0.34
mRS 0–4 at 12 months, n (%)	12 (31.6)*	7 (35)	5 (27.8)**	0.74

*T* temperature, *SD* standard deviation, *mRS* modified Rankin Scale. *n = 38, **n = 18

### Impact of temperature load and other factors on lethality at 12 months

The univariate logistic regression analysis revealed that the temperature load during the first 96 h after hemicraniectomy had no significant effect on 12 months lethality the cohort (OR 0.988 [95%-CI: 0.971–1.003]; *p* = 0.14). A clinical more meaningful increase of the temperature load of 10 °C*hours showed a comparable trend for lower case fatality rate (OR 0.884[95%-CI 0.742–1.032]; *p* = 0.13). A higher temperature deviation did also not increase the risk for lethality at 12 months (Table 3). Factors associated with a statistically significant higher risk for patients to decease within 12 months after stroke onset were higher patient age, the affection of the dominant hemisphere, and a shorter duration form stroke onset to decompressive surgery, respectively.

**Table 3:** Univariate regression analyses. Odds ratios for 12 months mortality

	Odds ratio	95%-CI	*p* value
Patient age	1.098	1.013–1.215	*0.022*
NIHSS score	0.994	0.899–1.101	0.903
Dominant hemisphere	6.818	1.437–50.512	*0.014*
Time to decompressive surgery	0.925	0.844–0.991	*0.022*
Temperature load (96 h, >36.5 °C; °C x hours)	0.988	0.971–1.003	0.122
Temperature deviation (96 h)	1.820	0.037–81.408	0.755

All *p* values in italics are below 0.05

Explorative multivariate regression modelling showed that the factors temperature load and temperature deviation, included with the factor age in the final model, did not significantly impact the probability for death at 12 months (temperature load: OR 0.986 [95%-CI: 0.967–1.002], *p* = 0.12; temperature deviation: OR 5.483 [95%-CI:0.072–478.958], *p* = 0.44; age: OR 1.116[1.020–1.262], *p* = 0.04; Hosmer-Lemeshow Test: C = 5.95; *p* = 0.65).

## Discussion

Decompressive surgery is an effective treatment for patients with space-occupying MCA infarction, but still a third of surgically treated patients die within 12 months, mainly during the intra-hospital treatment phase. Intensive care measures to reduce early lethality after decompressive surgery include body temperature management, but treatment strategies vary between centers.

In our single-center study, we analysed 40 stroke patients with hemicraniectomy, treated with normothermia according to a pre-specified temperature management protocol based on antipyretics and physical measures. This treatment strategy resulted in a low mean temperature load above 36.5 °C within the 96 h observational period after hemicraniectomy. Temperature load and temperature deviation were not predictive for 12 months lethality in our cohort.

Therapeutic hypothermia has been suggested as treatment option for space-occupying MCA infarction and was applied in several centers during the last 20 years, mainly in addition to decompressive surgery. Recent studies showed conflicting results for hypothermia in surgically treated stroke patients. In a small randomized trial with 33 patients, hypothermia (33 °C–34 °C) did not reduce mortality but survivors showed more often a better functional outcome if they were treated with hypothermia. A larger randomized controlled trial (DEPTH-SOS) was terminated for safety reasons and showed no reduction of early mortality in patients treated with hypothermia (33 °C–34 °C) compared to patients treated with standard care after decompressive surgery, but showed a higher rate of severe adverse events at 12 months (77% vs. 42%) [18]. Furthermore, no beneficial effects of hypothermia were found in a study evaluating treatment outcomes of a single-center cohort (hypothermia treatment [33 °C–34 °C] additionally to decompressive surgery) compared with outcomes of patients of randomized hemicraniectomy trials (standard treatment additionally to decompressive surgery) [17].

Maintaining normothermia after decompressive surgery was suggested as an alternative treatment goal to further reduce lethality in patients with space-occupying infarction [19]. Normothermia can be achieved either by prophylactic normothermia, e.g. with temperature management systems, or by reactive temperature management strategies. In our single-center cohort, a reactive strategy was used to achieve normothermia, excluding patients treated with temperature management systems.

The baseline characteristics of the cohort were comparable with the characteristics of hemicraniectomy trial samples, including stroke severity, involvement of the dominant hemisphere, and time to surgery. Median time to surgery was 29.5 h our single-center cohort, compared with 20.5 to 41 h in recent randomized controlled trials [2, 3, 20, 21]. Mean pre-operative body temperature was 36.6 °C in our cohort and 36.9 °C in the hemicraniectomy cohort of the pooled analysis by Vahedi et al. [1].

Postoperative mean temperature course in our cohort was characterized by a minimal postoperative temperature decline from pre-operative normothermia observed in most patients. This was followed by a moderate rise to a maximum of the mean temperature of about 37.2 °C within 24 h, and mild hyperthermia between 36.8 °C and 37.2 °C for the remaining observation period. Individual doses of acetaminophene used for temperature control were below the maximum daily dose permitted. Dual antipyretic therapy was applied in half the patients, and additional metamizole was applied in low doses. This implicates that in our cohort body temperatures below 37.5 °C were achieved for most patients during the post-operative observation period with relatively low doses of antipyretics.

Intrahospital lethality was 17% in our cohort, which is comparable to the data for ICU patients with ischemic stroke (18%) from an earlier large case series [22]. The long-term fatality rate in our cohort, including patients up to 80 years old, was 31.1% at 12 months. This is comparable to the results of previous controlled trials with mortality rates of 21% and 43% at 12 months [2, 3].

Temperature load >36.5 °C and temperature deviation within 96 h after hemicraniectomy were no independent predictors of long term mortality in our cohort. This can be explained in part by the low overall temperature load and the low to moderate temperature deviations observed. Due to the sample size, other factors potentially influencing mortality, e.g. infection rates or use of antibiotics, were not tested by multivariate analyses. Early infection rates and infection-related parameters such as leucocytosis were not often reported in recent trials for hemicraniectomy. In the DECIMAL cohort, 25% of patients had pneumonia and 10% had a urinary tract infection during the first week. In a large cohort of neurocritical care patients, infection rates (pneumonia, urinary tract infection) ranged from 13% to 33% [22]. Many patients with space-occupying stroke develop dysphagia and a reduced level of consciousness, leading to a high risk of aspiration despite prophylactic measures like body positioning and fasting. The high rate of early antibiotic use in our cohort implies a lower therapeutic threshold in these stroke patients, partially due to observed clinical signs of respiratory infection and signs of systemic inflammation (leukocytosis [90% of patients] and/or fever [65% of patients]).

An alternative to reactive normothermia management is the application of prophylactic normothermia, e.g. by endovascular or surface TMS. With the use of TMS devices a defined target temperature (including normothermia) can constantly achieved without relevant temperature deviations [23–25]. As temperature deviations in space-occupying infarction can lead to clinical relevant volume effects known as rebound phenomenon, this might be an important advantage of prophylactic normothermia using TMS compared to reactive temperature management strategies. On the other hand, the use of endovascular TMS can be associated with local or systemic infections or thrombosis, and body surface TMS can induce skin irritations.

Randomized controlled trials in patients with space-occupying infarction comparing a reactive normothermia management with prophylactic normothermia using TMS are lacking. Recently, the Impact of Fever Prevention in Brain Injured Patients (INTREPID) trial was initiated to asses the impact of fever prevention on fever burden and neurologic outcomes in stroke patients (ClinicalTrials.gov Identifier: NCT02996266). The fever prevention group in this trial will be treated by a body surface TMS and will be compared to patients treated according to a step-wise fever management algorithm, consisting primarily of intermittent antipyretics and cooling blankets. This algorithm is comparable to the temperature management protocol used in our cohort, which led to a mean temperature between 36.8 °C and 37.2 °C. Given the relatively small temperature difference between this observed mean temperature and a potential target temperature between 36.0 °C or 37.0 °C for prophylactic TMS normothermia, a randomized trial in patients with space-occupying MCA infarction would probably need a comparable large sample size like the INTREPID trial (*n* = 1176) to detect a possible, clinically meaningful difference between the two treatment strategies.

Furthermore, other aspects of temperature management deserve attention in future studies, e.g. the definition of normothermia, the threshold of fever, the duration of targeted temperature management, or the adverse effects of antipyretic treatments [26].

Our study has several strengths and limitations. We addressed our question analysing a well characterized cohort of patients with space-occupying MCA infarction treated with hemicraniectomy. Although reflecting practice outside clinical trials, the observed generally low temperature load above 36.5 °C suggests that temperature management with a simple protocol is feasible in clinical routine and that temperature management systems might be needed only in selected patients. Our data might therefore be used to estimate the sample sizes needed for future randomized trials comparing different temperature management strategies after decompressive surgery.

Given the design as an observational study, we did not compare temperature management strategies or protocols. The observed partial non-adherence to the normothermia protocol, e.g. use of antipyretic drugs in some patients below the pre-specified temperature level of 37.5 °C, is mainly attributable to its use in daily clinical routine. Another important limitation is the small number of patients analysed, which limits the possibility to identify associations of relevant variables with clinical outcomes. We evaluated only a limited number of factors relevant for the functional outcome of stroke ICU patients. Furthermore, we did not include safety measures in our analyses: antipyretics can be associated with hepatic and renal toxicity and increase the risk of bleeding [27, 28].

## Conclusions

Temperature management in stroke patients after decompressive surgery to achieve normothermia using a pre-specified protocol resulted in mild hyperthermia between 36.8 °C and 37.2 °C and a low overall temperature load. Functional outcome and mortality in our cohort at 12 months were comparable to outcomes reported for surgically treated patients of randomized hemicraniectomy trials.

Temperature load and temperature deviation were not independently predictive for long term mortality in our cohort of 40 stroke patients who underwent decompressive surgery. Future prospective studies on larger cohorts comparing different strategies for normothermia, including temperature management systems, are needed.

## Additional file

Additional file 1: Figure S1. The figure shows the temperature course of patients with highest (left) and lowest (right) temperature deviations during the 96 h observation period after hemicraniectomy. (DOC 151 kb)

## Abbreviations

TL: Temperature load
TMS: Temperature management system
