# Supplementary material for: Normothermia after decompressive surgery for space-occupying middle cerebral artery infarction: a protocol-based approach
Source: BMC Neurol. 2017 Dec 4;17:205. doi: 10.1186/s12883-017-0988-x (PMC5715533; doi:10.1186/s12883-017-0988-x)
Supplement: Additional file 1: Figure S1. — The figure shows the temperature course of patients with highest (left) and lowest (right) temperature deviations during the 96 h observation period after hemicraniectomy. (DOC 151 kb) [file 12883_2017_988_MOESM1_ESM.doc]

**Supplemental Figure**

The figure shows the temperature course of patients with highest (left) and lowest (right) temperature deviations during the 96 hour observation period after hemicraniectomy.


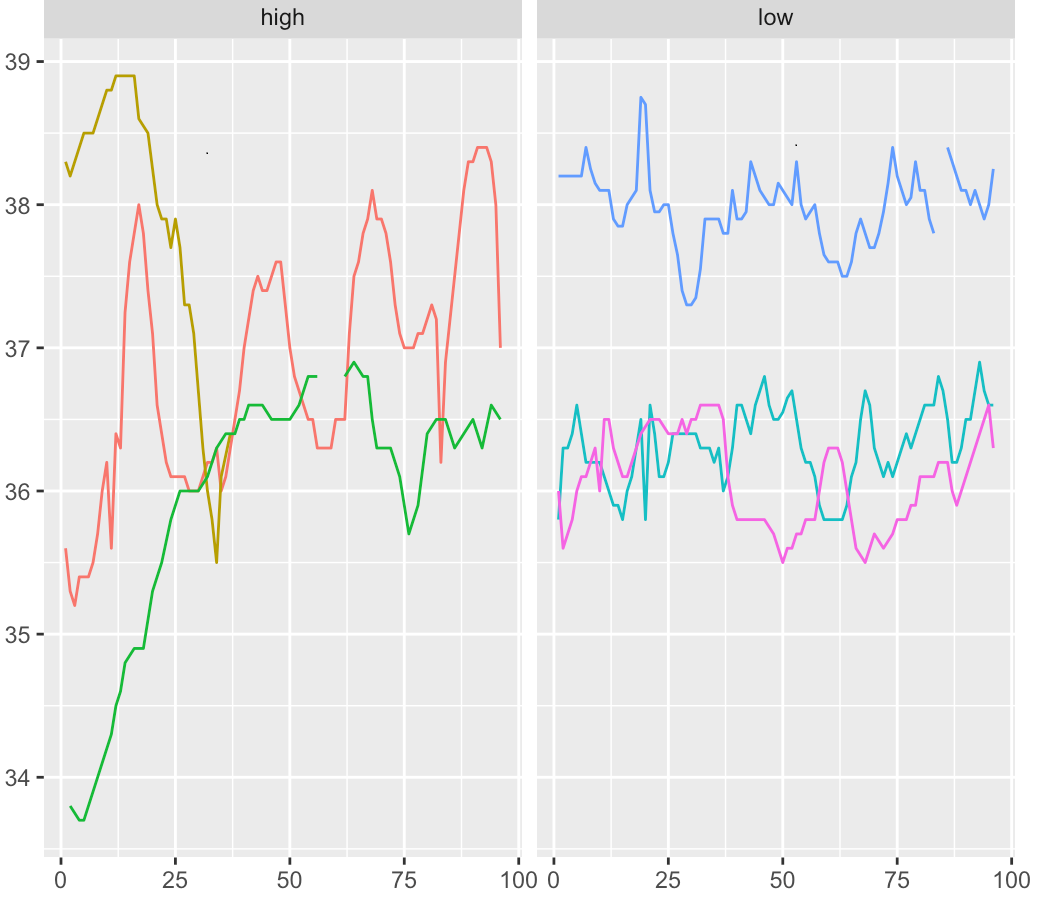


**T (°C)**

**t (h)**

**T**, temperature; **t**, time after hemicraniectomy
